# Impact of Cognitive Abilities and Prior Knowledge on Complex Problem Solving Performance – Empirical Results and a Plea for Ecologically Valid Microworlds

**DOI:** 10.3389/fpsyg.2018.00626

**Published:** 2018-05-08

**Authors:** Heinz-Martin Süß, André Kretzschmar

**Affiliations:** ^1^Institute of Psychology, Otto-von-Guericke University Magdeburg, Magdeburg, Germany; ^2^Hector Research Institute of Education Sciences and Psychology, University of Tübingen, Tübingen, Germany

**Keywords:** complex problem solving, microworlds, minimally complex systems, intelligence, investment theory, knowledge assessment, working memory, Brunswik symmetry

## Abstract

The original aim of complex problem solving (CPS) research was to bring the cognitive demands of complex real-life problems into the lab in order to investigate problem solving behavior and performance under controlled conditions. Up until now, the validity of psychometric intelligence constructs has been scrutinized with regard to its importance for CPS performance. At the same time, different CPS measurement approaches competing for the title of the best way to assess CPS have been developed. In the first part of the paper, we investigate the predictability of CPS performance on the basis of the Berlin Intelligence Structure Model and Cattell’s investment theory as well as an elaborated knowledge taxonomy. In the first study, 137 students managed a simulated shirt factory (*Tailorshop*; i.e., a complex real life-oriented system) twice, while in the second study, 152 students completed a forestry scenario (*FSYS*; i.e., a complex artificial world system). The results indicate that reasoning – specifically numerical reasoning (Studies 1 and 2) and figural reasoning (Study 2) – are the only relevant predictors among the intelligence constructs. We discuss the results with reference to the Brunswik symmetry principle. Path models suggest that reasoning and prior knowledge influence problem solving performance in the *Tailorshop* scenario mainly indirectly. In addition, different types of system-specific knowledge independently contribute to predicting CPS performance. The results of Study 2 indicate that working memory capacity, assessed as an additional predictor, has no incremental validity beyond reasoning. We conclude that (1) cognitive abilities and prior knowledge are substantial predictors of CPS performance, and (2) in contrast to former and recent interpretations, there is insufficient evidence to consider CPS a unique ability construct. In the second part of the paper, we discuss our results in light of recent CPS research, which predominantly utilizes the minimally complex systems (MCS) measurement approach. We suggest ecologically valid microworlds as an indispensable tool for future CPS research and applications.

## Introduction

People are frequently confronted with problems in their daily lives that can be characterized as complex in many aspects. A subset of these problems can be described as interactions between a person and a dynamic system of interconnected variables. By manipulating some of these variables, the person can try to move the system from its present state to a goal state or keep certain critical variables within tolerable ranges. Problems of this kind can be simulated using computer models (aka microworlds), offering an opportunity to observe human behavior in realistic problem environments under controlled conditions.

The study of human interaction with complex computer-simulated problem scenarios has become an increasingly popular field of research in numerous areas of psychology over the past four decades. For example, computer models have been built to simulate the job of a small-town mayor (Dörner et al., 1983), a production plant operator (Bainbridge, 1974; Morris and Rouse, 1985), a business manager (Putz-Osterloh, 1981; Wolfe and Roberts, 1986), a coal-fired power plant operator (Wallach, 1997), and a water distribution system operator (Gonzalez et al., 2003). Real-time simulations have put users in the role of the head of a firefighting crew (Brehmer, 1986; Rigas et al., 2002) or an air traffic controller (Ackerman and Kanfer, 1993). In experimental psychology, research on complex problem solving (CPS) has sought to formally describe simulations (e.g., Buchner and Funke, 1993; Funke, 1993), the effects of system features on task difficulty (e.g., Funke, 1985; Gonzalez and Dutt, 2011), the role of emotions (e.g., Spering et al., 2005; Barth and Funke, 2010), and the effects of practice and training programs (e.g., Kluge, 2008b; Kretzschmar and Süß, 2015; Goode and Beckmann, 2016; Engelhart et al., 2017; see also Funke, 1995, 1998). Differential and cognitive psychology research has investigated the psychometrical features of CPS assessments (e.g., Rigas et al., 2002), the utility of computational models for explaining CPS performance (e.g., Dutt and Gonzalez, 2015), the relationship between CPS performance and cognitive abilities (e.g., Wittmann and Süß, 1999), and its ability to predict real-life success criteria (e.g., Kersting, 2001). For detailed summaries of different areas of CPS research, see Frensch and Funke (1995) and Funke (2006).

Meanwhile, many researchers have moved away from complex real life-oriented systems (CRS) to complex artificial world systems (CAS) in order to increase the psychometric quality of measures and to control for the effects of preexisting knowledge (e.g., Funke, 1992; Wagener, 2001; Kröner et al., 2005). This development ultimately culminated in the minimally complex systems (MCS) approach (Greiff et al., 2012), also known as the multiple complex systems approach (e.g., Greiff et al., 2015a). This approach has recently become prominent in educational psychology (e.g., Greiff et al., 2013b; Sonnleitner et al., 2013; Kretzschmar et al., 2014; OECD, 2014; Csapó and Molnár, 2017). In addition, this shift has led to the question of what are and are not complex problems, with some researchers questioning the relevance of MCS as a tool for CPS research and the validity of the conclusions drawn from them (e.g., Funke, 2014; Dörner and Funke, 2017; Funke et al., 2017; Kretzschmar, 2017).

Originally, simulated dynamic task environments were used to reproduce the cognitive demands associated with real-life problems in the laboratory (Dörner et al., 1983; Dörner, 1986). These environments have several features: (1) Complexity: Many aspects of a situation must be taken into account at the same time. (2) Interconnectivity: The different aspects of a situation are not independent of one another and therefore cannot be controlled separately. (3) Intransparency: Only some of the relevant information is made available to the problem solver. (4) Dynamics: Changes in the system occur without intervention from the agent. (5) Polytely: The problem solver must sometimes pursue multiple and even contradictory goals simultaneously. (6) Vagueness: Goals are only vaguely formulated and must be defined more precisely by the problem solver. Whereas older microworlds featured all of these characteristics to a considerable extent, more recent approaches such as MCS have substituted complexity and ecological validity (i.e., the simulation’s validity as a realistic problem-solving environment allowing psychological statements to be made about the real world; see Fahrenberg, 2017) for highly reliable assessment instruments by simulating tiny artificial world relationships (e.g., Greiff et al., 2012; Sonnleitner et al., 2012).

The present paper is divided into two parts. In the first part, we deal with one of the oldest but still an ongoing issue in the area of CPS research: the cognitive prerequisites of CPS performance. In two different studies, we used microworlds (CRS and CAS) to empirically investigate the impact of cognitive abilities (i.e., intelligence and working memory capacity) and prior knowledge on CPS performance. In doing so, we considered the impact of the Brunswik symmetry principle, which effects the empirical correlations between hierarchical constructs (e.g., Wittmann, 1988). Integrating our results with previous CPS research, we review the basis and empirical evidence for ‘complex problem solving ability’ as a distinct cognitive construct. In the second part of the paper, we discuss our approach and results in light of recent problem solving research, which predominantly utilizes the MCS approach. Finally, we conclude with some recommendations for future research on CPS and suggest ecologically valid microworlds as tools for research and applications.

## Part I: Empirical Investigation of the Cognitive Prerequisites of Complex Problem Solving Performance

### Intelligence and Complex Problem Solving

At the beginning of complex problem solving (CPS) research, CPS pioneers raised sharp criticisms of the validity of psychometric intelligence tests (Putz-Osterloh, 1981; Dörner et al., 1983; Dörner and Kreuzig, 1983). These measures, derisively referred to as “test intelligence,” are argued to be bad predictors of performance on partially intransparent, ill-defined complex problems. In contrast to simulated scenarios, intelligence test tasks are less complex, static, transparent, and well-defined problems that do not resemble most real-life demands in any relevant way. Zero correlations between intelligence measures and CPS performance were interpreted as evidence of the discriminant validity of CPS assessments, leading to the development of a new ability construct labeled complex problem solving ability or operative intelligence (Dörner, 1986). However, no evidence of the convergent validity of CPS assessments or empirical evidence for their predictive validity with regard to relevant external criteria or even incremental validity beyond psychometric intelligence tests have been presented.

By now, numerous studies have investigated the relationship between control performance on computer-simulated complex systems and intelligence. Whereas Kluwe et al. (1991) found no evidence of a relationship in an older review, more recent studies have found correlations that are substantial but still modest enough to argue in favor of a distinct CPS construct (e.g., Wüstenberg et al., 2012; Greiff et al., 2013b; Sonnleitner et al., 2013). In a more recent meta-analysis, Stadler et al. (2015) calculated the overall average effect size between general intelligence (g) and CPS performance to be *r* = 0.43 (excluding outliers, *r* = 0.40), with a 95% confidence interval ranging from 0.37 to 0.49. The mean correlation between CPS performance and reasoning was *r* = 0.47 (95% CI: 0.40 to 0.54). The relationship with g was stronger for MCS (*r* = 0.58) than CRSs (*r* = 0.34)^1^. From our point of view, this difference results from the higher reliability of MCS but also a difference in cognitive demands. MCS are tiny artificial world simulations in which domain-specific prior knowledge is irrelevant. Complex real life-oriented tasks, however, activate preexisting knowledge about the simulated domain. This knowledge facilitates problem solving; in some cases, the problems are so complex that they cannot be solved at all without prior knowledge (e.g., Hesse, 1982).

The main issues with many complex real life-oriented studies that investigated the relation between intelligence and CPS performance concern the ecological validity of the simulations and the psychometric quality of the problem-solving performance criteria. This often leads to much larger confidence intervals in their correlations with intelligence compared to minimal complex tasks (Stadler et al., 2015). When the goals of a simulation are multiple and vaguely defined, the validity of any objective criterion is questionable since it might not correspond to the problem solver’s subjective goal. However, people are unlikely to face a single, well-defined goal in real-life problems, limiting the ecological validity of such systems – despite the fact that a well-defined goal is a necessary precondition for assessing problem solving success in a standardized way, which is necessary in order to compare subjects’ performance. Moreover, single problem solving trials produce only “single act criteria” (Fishbein and Ajzen, 1974), criticized as “one-item-testing” (e.g., Wüstenberg et al., 2012), the reliability of which is severely limited. Performance scores must be aggregated via repeated measurements to increase the proportion of reliable variance that can be predicted (e.g., Wittmann and Süß, 1999; Rigas et al., 2002). The MCS has implemented these steps, resulting in strong reliability estimates (e.g., Greiff et al., 2012; Sonnleitner et al., 2012).

Another crucial issue with regard to the relation between intelligence and CPS performance is the operationalization of intelligence. Numerous prior studies have used a measure of general intelligence (*g*) to predict problem solving success. Since *g* is a compound of several more specific abilities, *g* scores comprise variance in abilities relevant to complex problem solving as well as variance in irrelevant abilities. According to Wittmann’s (1988) multivariate reliability theory and the Brunswik symmetry principle (see also Wittmann and Süß, 1999), this results in an asymmetric relationship between predictor and criterion, attenuating their correlation. More specific subconstructs of intelligence might be more symmetrical predictors because they exclude irrelevant variance. In our view, controlling complex systems requires a great deal of reasoning ability (e.g., Süß, 1996; Wittmann and Süß, 1999; Kröner et al., 2005; Sonnleitner et al., 2013; Kretzschmar et al., 2016, 2017). Inductive reasoning is required to detect systematic patterns within the ever-changing system states and develop viable hypotheses about the system’s causal structure. Deductive reasoning is necessary to infer expectations about future developments from knowledge of causal connections and deduce more specific goals from higher-order goals. Abilities such as perceptual speed (except in real-time simulations), memory, and verbal fluency, meanwhile, should be less relevant for success in complex problem solving. In this sense, it is an open question in CPS research whether WMC, as a more basic ability construct (e.g., Süß et al., 2002; Oberauer et al., 2008), is a more symmetrical predictor of CPS performance than reasoning (for an overview of previous findings, see Zech et al., 2017).

In summary, a substantial correlation between intelligence and CPS performance measured with real life-oriented microworlds can be expected if (1) sufficient reliability of the CPS measures is ensured (e.g., aggregation via repeated measures), and (2) the best symmetrical intelligence construct is used (e.g., reasoning instead of general intelligence or perceptual speed).

### Knowledge and Complex Problem Solving

In addition to the debate about intelligence’s contribution to complex problem solving, many researchers have pointed out the significance of knowledge for the successful control of complex systems (e.g., Bainbridge, 1974; Dörner et al., 1983; Chi et al., 1988; Goode and Beckmann, 2010; Beckmann and Goode, 2014). Expert knowledge is sometimes claimed to be the only important predictor of real-life problem solving success (Ceci and Liker, 1986), while others point out that both intelligence and knowledge contribute substantially to predicting job performance (Schmidt, 1992), which certainly includes complex problem solving.

Scenarios that accurately simulate real-world relationships provide an opportunity to draw on preexisting knowledge about the part of reality being simulated. That being said, a simulation never is exactly equivalent to what the problem solver has experienced before. Experts in a domain can make use of their knowledge to operate a simulation within that domain, but they are not automatically experts in the simulated scenario. The application of domain knowledge to the simulation requires a considerable amount of transfer. Following Cattell’s investment theory (Cattell, 1987), we assume that intelligence, and particularly reasoning, plays an important role in mediating this transfer. Therefore both, intellectual abilities, particularly reasoning and prior knowledge of the simulated domain, should be powerful predictors of complex problem solving success, although the effect of intelligence has been found to be mainly indirect, mediated through knowledge (Schmidt et al., 1986; Schmidt, 1992).

The knowledge relevant for successfully controlling a complex system can be differentiated conceptually on two dimensions. First, knowledge about the system can be distinguished from knowledge about appropriate actions. System knowledge is knowledge about the features and structure of a system, such as what variables it consists of, how these variables are related, and what kind of behaviors the system tends to exhibit. Action-related knowledge is knowledge about what to do in order to pursue a given goal. In contrast to system knowledge, action knowledge is always bound to a specific goal. Studies by Vollmeyer et al. (1996) provided evidence for the distinction between system knowledge and action knowledge: Participants who acquired knowledge about a system during an exploration phase with or without a given goal performed equally well on a subsequent test trial with the same goal. However, the group which had not been given a specific goal during the exploration phase outperformed the group with the specific goal on a test with a new goal. Presumably, the specific goal group had learned mainly action knowledge, whereas the other group had acquired more system knowledge, which was then transferable to new goals.

A second distinction, independent of the first, exists between declarative and procedural knowledge. Declarative knowledge is knowledge that a person can represent symbolically in some way – verbally, graphically or otherwise. Declarative knowledge can be expressed as accurate answers to questions. Procedural knowledge, on the other hand, can be expressed only through accurate performance. The distinction between declarative and procedural knowledge is based on the conceptual difference between “knowing that” and “knowing how” (Ryle, 1949).

While system knowledge and action knowledge differ in content, declarative and procedural knowledge are different forms of knowledge. Therefore, the two dimensions can be conceived of as orthogonal. System knowledge and action knowledge can both be declarative: A person can talk about which variables are causally related to which other variables, but also about what to do in order to keep the system stable. Similarly, both system knowledge and action knowledge can also be procedural: Knowing how to stabilize a system without being able to express it is procedural action knowledge. Being able to mentally simulate a system or diagnose what variable is causing a disturbance without being able to give a full verbal account of the reasons is indicative of procedural system knowledge. Several studies have found that people do not improve their problem-solving performance in controlling or repairing complex systems after receiving instructions in the form of declarative system knowledge (e.g., Morris and Rouse, 1985; Kluge, 2008b; but see Goode and Beckmann, 2010), and declarative knowledge sometimes is not correlated with problem solving performance (e.g., Berry and Dienes, 1993). Therefore, we must consider the possibility that procedural knowledge is part of the relevant knowledge base that guides a person’s actions within complex dynamic environments.

In summary, prior domain knowledge must be considered as an additional substantial predictor of CPS performance. However, differentiating between different types of knowledge is necessary in order to explain CPS performance. In addition, different semantic embeddings (i.e., CRS vs. CAS) have different demands with regard to preexisting knowledge.

### The Present Study

**FIGURE 1:**
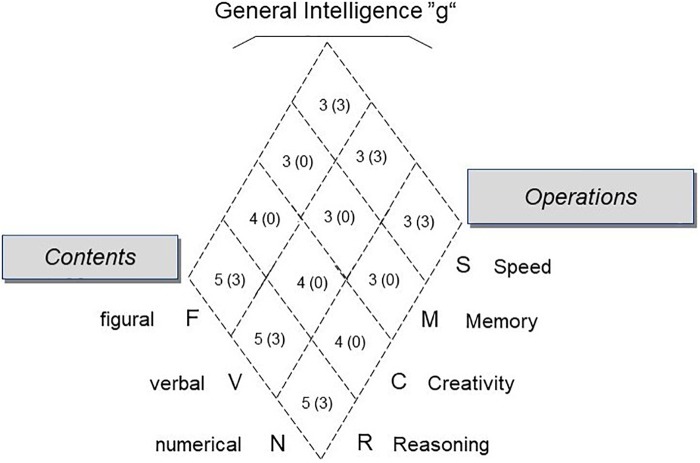
The Berlin Intelligence Structure Model (BIS), and the number of tasks for each cell applied in Study 1 (in brackets, Study 2). In the BIS, four operation ability constructs are crossed with three content constructs, yielding twelve cells. On a higher level of aggregation, general intelligence integrates the primary factors for each facet.

The first goal of the two studies presented in this paper was to test the hypothesized criterion validity of reasoning in predicting problem solving performance in complex dynamic tasks. In addition, considering the Brunswik symmetry principle (Wittmann, 1988), we explored the predictive validity of additional more specific or more general intelligence constructs. Our investigation was based on the Berlin Intelligence Structure Model (BIS), a hierarchical and faceted model of intelligence (Jäger, 1982, 1984; for a detailed description in English, see Süß and Beauducel, 2015). The BIS differentiates intellectual abilities along two facets. The operation facet comprises four abilities: Reasoning (R) includes inductive, deductive and spatial reasoning and is equivalent to fluid intelligence (Gf). Creativity (C) refers to the ability to fluently produce many different ideas. Memory (M) refers to the ability to recall lists and configurations of items a few minutes after having learned them (episodic memory), whereas speed (S) refers to the ability to perform simple tasks quickly and accurately (perceptual speed). The second facet is postulated to include three content-related abilities: verbal (V), numerical (N) and figural-spatial (F) intelligence. Cross-classifying the four operational and three content abilities results in 12 lower-order cells. In addition, general intelligence is conceptualized as an overarching factor (**Figure 1**). For summaries of the validity and scope of the BIS, see the handbook for the BIS Test (Jäger et al., 1997) as well as Süß and Beauducel (2005, 2015).

In the second study, we included WMC as an additional predictor. Working memory is considered the most important cognitive resource for complex information processing, which includes reasoning (e.g., Kyllonen and Christal, 1990; Süß et al., 2002; Conway et al., 2003), language comprehension (e.g., King and Just, 1991), and math performance (e.g., Swanson and Kim, 2007). Consequently, previous research has found a significant relation between WMC and CPS (e.g., Wittmann and Süß, 1999; Bühner et al., 2008; Schweizer et al., 2013; Greiff et al., 2016). However, whether the more basic construct (i.e., WMC) is a stronger symmetrical predictor of CPS than reasoning from the perspective of the Brunswik symmetry principle (Wittmann, 1988) is not clear (for an overview, see Zech et al., 2017). For example, Wittmann and Süß, 1999 demonstrated that WMC has incremental validity in predicting CPS performance beyond intelligence. Bühner et al. (2008) could not confirm this result, but their study relied upon narrow operationalizations.

The second goal of the two studies presented in this paper was to investigate the relation between knowledge and complex problem solving performance. We attempted to measure knowledge about complex systems in several categories. We focused on declarative knowledge in the form of both system knowledge and action knowledge because assessing declarative knowledge is straightforward. We also attempted to measure procedural knowledge, despite the fact that no evidence has ever been put forward that responses to complex problem-solving tests exclusively reflect procedural knowledge and not declarative knowledge. Based on Cattell’s investment theory (Cattell, 1987), we assumed that knowledge represents invested intelligence and examined whether the predictive effect of intelligence on CPS performance is completely mediated by prior knowledge.

We applied a CRS (i.e., a microworld with a realistic semantic embedding) in the first study, whereas we used a CAS (i.e., a microworld with an artificial semantic embedding) in the second study. Hence, the importance of preexisting knowledge with regard to CPS performance should differ between the two studies.

## Study 1

In the first study, we used a complex real life-oriented simulation to examine the criterion validity of intelligence, particularly reasoning, and prior knowledge for control performance in a simulated shirt factory (*Tailorshop*). As we used a very comprehensive assessment of intelligence and knowledge, we were also interested in exploring the predictive validity of additional, more specific constructs in order to investigate the influence of the Brunswik symmetry principle (Wittmann, 1988) on the relation between intelligence, knowledge and CPS performance.

### Method

#### Participants

One hundred and thirty-seven students from 13 high schools in Berlin took part in the experimental study in 1990 (Süß et al., 1991). They had all participated in a similar study 1 year before in which they had taken prior versions of the BIS Test and the knowledge tests and had explored the *Tailorshop* system (Süß et al., 1993a,b). Their mean age was 17.6 years (*SD* = 0.67), and 40.9% were female. The participants were fully informed about the study and the voluntary nature of their participation, and anonymity was guaranteed. Written informed consent was obtained from school principals and the state school board. Subjects who withdrew from the study were required to attend other school lessons. Both Berlin studies were published in German only; a full report including the longitudinal results can be found in Süß (1996). In this paper, we report the results of the second Berlin study (here labeled Study 1) to make the results available for international readers and to discuss the two studies in the light of recent developments in CPS research.

#### Materials

##### Problem solving

**FIGURE 2:**
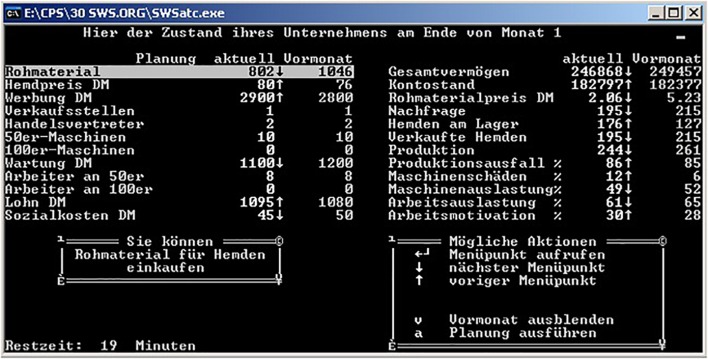
Screenshot of the exploration phase of the Tailorshop system as applied in Study 1.

**FIGURE 3:**
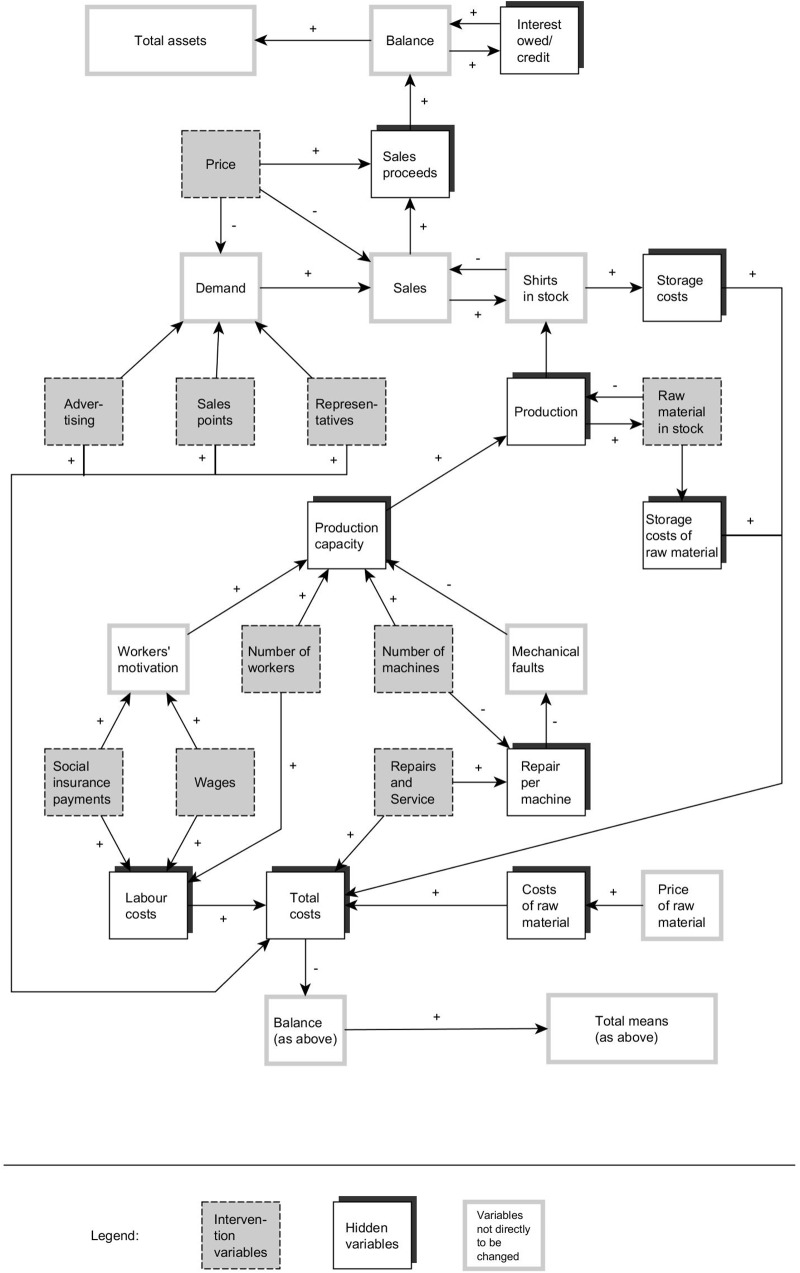
The causal structure of the Tailorshop system.

An extended version of the *Tailorshop* system (Funke, 1983; Danner et al., 2011), originally designed by D. Dörner and first used in a published study by Putz-Osterloh (1981), was applied as a *CRS* (Süß and Faulhaber, 1990). Additional minor modifications were made in the system to resolve issues with the validity of the problem-solving score that had become apparent in the study conducted 1 year before (Süß et al., 1993a,b). *Tailorshop* is a computer simulation of a shirt factory. The system has 27 variables: 10 are exogenous variables that can be manipulated directly, and 17 are endogenous variables computed by the simulation. **Figure 2** provides a screenshot of the system, and **Figure 3** an overview of the variables and their interconnections.

The system was run on a personal computer. All variables were presented in a single menu, and the values of exogenous variables could be selected via a pull-down menu. After planning all decisions, the operator ran the simulation for one virtual month. A complete trial consisted of twelve simulation cycles corresponding to 1 year of management. To obtain two independent indicators of problem solving success, participants worked on two versions of *Tailorshop* with different starting values corresponding to different shirt factories and different economic conditions. Problem solving performance was measured by participants’ total assets after 12 simulated months. Since the distribution of raw scores deviated considerably from a normal distribution, we transformed them into rank scores and aggregated participants’ ranks from the two simulation runs into one total score.

##### Intelligence test

To assess intellectual abilities, we used a prior version of the BIS Test (Jäger et al., 1997; for a full English description see Süß and Beauducel, 2015; for prior test versions see Süß, 1996). This test consists of three to five different tasks for each of the 12 cells in the matrix structure of the BIS. Each task assigned to a cell in the model is used to measure one operation ability as well as one content ability. The four operation abilities are thus measured with scales consisting of 9–15 tasks each and balanced over the three content categories. Analogously, content abilities are measured with scales consisting of 15 tasks across the four different operation abilities. Thus, the same variables are used in different ways for different scales. The scales for one facet are built by aggregating variables that are distributed in a balanced way over the other facet. This suppresses unwanted variance, i.e., the variance associated with factors from the other facet (Wittmann, 1988). However, the scores for operation abilities and content abilities are not statistically independent. An indicator of general intelligence is built by aggregating either the operation scores or content scores.

##### Knowledge tests

Preexisting general economics knowledge was assessed with an age-normed economics test (Deutsche Gesellschaft für Personalwesen [DGP], 1986, with a few questions added from the economics test from Krumm and Seidel, 1970)^2^. The questionnaire consisted of 25 multiple-choice items on the meaning of technical terms from the domain of economics.

A new test was developed to assess system-specific knowledge about *Tailorshop* (Kersting and Süß, 1995). This test had two parts, one for system knowledge and one for action knowledge.

System knowledge refers to knowledge about features of individual variables (e.g., development over time, degree of connectedness with other variables) and about relationships between variables in a system. The system knowledge part of the test was developed in accordance with test construction principles for optimizing content validity (Klauer, 1984; Haynes et al., 1995). It consisted of three scales:

(1) Multiple choice questions about the connections between two variables. One out of six statements in the following form had to be selected as correct:
  (a) An increase in variable X increases variable Y.
  (b) An increase in variable X decreases variable Y.
  (c) An increase in variable Y increases variable X.
  (d) An increase in variable Y decreases variable X.
  (e) Variable X and variable Y interact, that is, they both depend on one another.
  (f) (a) through (e) are false.
  There were 20 questions of this type.

(2) Questions about hypotheses concerning single variables: Participants had to evaluate statements about the regular behavior of individual system variables, e.g., “The price of shirts rises and falls by chance” (which is false) or “Production depends – among other factors – on my workers’ motivation, which in turn depends on the level of wages” (which is true). The scale consisted of 25 independent items.
(3) Arrow test for connections among multiple variables: Sets of four variables were represented by labeled boxes in a diamond-shaped arrangement. Participants had to draw arrows connecting the variables that had a direct causal connection in the system, and designate the direction of correlation with a plus or minus sign (as in **Figure 3**). Each of the six possible pairings in a set was counted as an independent item that was marked as either correct or incorrect, yielding a total of 42 items.

Action knowledge refers to knowledge about appropriate actions in a certain situation, given a certain goal. It was assessed in this study via two subtests. The test of declarative action knowledge presented “rules of thumb” for successfully managing the *Tailorshop* simulation, which had to be evaluated as correct or incorrect. Half of the 12 rules were correct, i.e., they were helpful in obtaining high total assets within 12 months, while the other half were incorrect.

In the second subtest, participants were given a system state in the form of a screen display. They were given the goal of maximizing or minimizing a certain system variable, for example, minimizing the number of shirts in the store. They had to select which one out of six alternative decision patterns would be best-suited to reaching this goal in the next simulation cycle. This subtest consisted of six items with different system states, goals, and decision options. In contrast to the declarative questions, this task did not require participants to explicit declare rules for action. Instead, the rules governing their decision-making remained implicit, providing a good opportunity to capture task relevant procedural knowledge. Thus, we will refer to this subscale as procedural action knowledge.

Sum scores were built for each subtest and a total score was calculated by aggregating the subtest scores, weighted equally.

Each type of question was introduced by the experimenter with one or two examples. There was no time limit, but participants were instructed not to spend too much time on any single question.

#### Procedure

The students took tests on 2 days for 5–6 h each. On the first day, they worked on the BIS Test and the general economics test as well as some further questionnaires. Testing was done in groups of 20–30 in school classrooms. On the second day, participants were first introduced to the *Tailorshop* system via detailed instructions, including two standardized practice cycles guided by the experimenter. Afterward, the students in the sample were randomly divided into three groups, and two groups were given additional opportunities to acquire system-specific knowledge.^3^ Next, system-specific knowledge was assessed (time T1) by instructing participants to build hypotheses about *Tailorshop* on basis of their (superficial) experience with the system. Participants then tried to manage the *Tailorshop* twice for 12 simulated months. Finally, system-specific knowledge was tested again (time T2). The knowledge test took about 80 min the first time and about 60 min the second time. Each problem solving trial lasted about 50 min. The participants took these tests in smaller groups at the university’s computer lab.

### Results

We will first present the results of separate analyses of the relationship between problem solving performance and different groups of predictors. Then, we integrate all the variables into a path model. Ten participants had missing data for the economics knowledge test. Thus, we applied the full information maximum likelihood (FIML) procedure to account for the missing data. See **Table 1** for descriptive statistics and the full correlation matrix.

**Table 1:** Study 1: Means, standard deviations, and correlations.

Variable	*M*	*SD*	1	2	3	4	5	6	7	8	9	10	11	12	13	14	15	16	17	18	19
(1) BIS: g	-0.03	6.76																			
(2) BIS: Speed	-0.02	2.30	0.77**																		
(3) BIS: Memory	-0.02	2.36	0.66**	0.31**																	
(4) BIS: Creativity	0.00	2.22	0.66**	0.38**	0.21*																
(5) BIS: Reasoning	0.00	2.51	0.79**	0.53**	0.35**	0.35**															
(6) BIS: Verbal	0.00	2.60	0.78**	0.57**	0.52**	0.57**	0.59**														
(7) BIS: Figural	-0.03	2.55	0.83**	0.62**	0.52**	0.61**	0.64**	0.52**													
(8) BIS: Numerical	-0.00	3.12	0.84**	0.69**	0.56**	0.46**	0.69**	0.44**	0.55**												
(9) Know: General	0.02	1.77	0.10	-0.12	-0.01	0.20*	0.21*	0.11	0.14	0.01											
(10) Know: Dec. Sys. t1	1.81	0.44	0.24**	0.08	0.06	0.07	0.46**	0.18*	0.18*	0.23**	0.25**										
(11) Know: Dec. Act. t1	5.69	1.73	-0.00	0.04	-0.18*	0.07	0.06	-0.08	0.01	0.05	0.21*	0.11									
(12) Know: Pro. Act. t1	11.15	3.47	0.10	0.07	0.08	0.00	0.13	0.04	0.04	0.16	0.12	0.15	0.16								
(13) Know: Spec. Tot. t1	72.75	13.07	0.25**	0.10	0.04	0.10	0.46**	0.18*	0.18*	0.24**	0.30**	0.93**	0.28**	0.41**							
(14) Know: Dec. Sys. t2	1.87	0.40	0.30**	0.13	0.13	0.06	0.53**	0.21*	0.21*	0.31**	0.25**	0.83**	0.07	0.07	0.75**						
(15) Know: Dec. Act. t2	6.94	1.82	0.16	0.15	-0.01	0.03	0.29**	0.06	0.15	0.18*	0.15	0.27**	0.49**	0.18*	0.34**	0.24**					
(16) Know: Pro. Act. t2	11.83	3.41	0.07	0.06	0.08	-0.07	0.13	-0.05	0.08	0.13	0.14	0.17*	0.17*	0.54**	0.30**	0.17*	0.14				
(17) Know: Spec. Tot. t2	76.84	12.37	0.32**	0.18*	0.12	0.05	0.55**	0.19*	0.24**	0.35**	0.28**	0.80**	0.18*	0.22*	0.79**	0.94**	0.38**	0.41**			
(18) CPS	138.00	72.58	0.22*	0.08	-0.03	0.22*	0.34**	0.11	0.16	0.25**	0.36**	0.43**	0.36**	0.24**	0.51**	0.37**	0.28**	0.29**	0.46**		
(19) Gender	1.41	0.49	-0.04	0.04	0.08	-0.05	-0.17	0.15	-0.04	-0.17	-0.42**	-0.32**	-0.15	-0.06	-0.33**	-0.38**	-0.14	-0.11	-0.38**	-0.35**	
(20) Age	17.55	0.67	-0.21*	-0.20*	-0.12	-0.08	-0.19*	-0.18*	-0.16	-0.17	0.09	-0.25**	-0.06	0.05	-0.21*	-0.19*	-0.22**	-0.02	-0.19*	-0.24**	-0.02


*^∗^ Indicates *p* < 0.05; ^∗∗^ indicates *p* < 0.01. *M* and *SD* are used to represent mean and standard deviation, respectively. BIS, Berlin Intelligence Structure Test; Know: General, general knowledge (economics); Know: Dec. Sys, declarative system knowledge; Know: Dec. Act., declarative action knowledge; Know: Pro. Act., procedural action knowledge; Know: Spec. Tot., total problem-specific knowledge; CPS, complex problem solving (*Tailorshop*); t1, measurement at the Time 1; t2, measurement at the Time 2.*

#### Complex Problem Solving and Intelligence

The parallel-test reliability of problem solving performance was *r* = 0.67 (*p* < 0.01). This indicates that the criterion measures had satisfactory reliability and justifies their aggregation into a single score. Two multivariate regressions were computed with the aggregated performance criterion, first with the four operation scales and then with the three content scales of the BIS as predictors. The results are summarized in **Table 2** (upper half, correlations in brackets).

Among the operation scales, reasoning (*r* = 0.34, *p* < 0.01) was as expected significantly correlated with problem-solving success, furthermore, creativity (*r* = 0.22, *p* = 0.01) as well. In the regression model, however, only reasoning had a significant beta weight (β = 0.43, *p* < 0.01). Among the content scales, only numerical intelligence had a significant beta weight (β = 0.22, *p* = 0.03). The proportion of variance accounted for by the operation scales was much higher than that accounted for by the content scales, despite the fact that the two groups of predictors consisted of the same items that had merely been aggregated in different ways. Building an overall aggregate for all BIS scales (BIS-g) only accounted for five percent of the criterion variance (*r* = 0.22, *p* = 0.01)^4^, compared to 15 percent with the four operation scales. In line with the Brunswik symmetry principle (Wittmann, 1988; Wittmann and Süß, 1999), this comparison shows the benefit of differentiating intellectual abilities into multiple components using a multi-faceted model. Taking the cell level of the BIS^5^ into account, numerical reasoning was the best and thus likely the most symmetrical predictor of *Tailorshop* performance (*r* = 0.36, *p* < 0.01).^6^ While the correlation between the numerical reasoning cell and the criterion was nearly the same as the correlation for reasoning, numerical reasoning was the better predictor given the substantially lower reliability of the cell score for numerical reasoning (Cronbach’s α = 0.77) compared to reasoning (1-year stability, *r* = 0.90, *p* < 0.01). Corrected for unreliability, the true correlation was *r* = 0.43. In summary, aggregating repeated measures increases the reliability and thus also the validity of the CPS performance score. However, the correlations are lower than for minimally complex tasks even on the most symmetrical level (*r* = 0.58), as reported in Stadler et al.’s (2015) meta-analysis.

#### Complex Problem Solving and Knowledge

Four scales representing prior knowledge (time T1) were used as predictors of problem solving success in the regression analysis. These were the general economics test and the three categories of knowledge represented in the system-specific knowledge test: declarative system knowledge (measured with three subtests), declarative action knowledge (measured with the rules of thumb), and procedural action knowledge (measured using the system-states task). General economics knowledge (β = 0.21, *p* < 0.01; *r*_zero-order_ = 0.36, *p* < 0.01), declarative system knowledge (β = 0.33, *p* < 0.01; *r*_zero-order_ = 0.43, *p* < 0.01), and declarative action knowledge (β = 0.26, *p* < 0.01; *r*_zero-order_ = 0.36, *p* < 0.01) were significantly associated with problem solving performance, whereas procedural action knowledge was not (β = 0.13, *p* = 0.07; *r*_zero-order_ = 0.24, *p* < 0.01). The latter might be in part due to the low reliability of the test, which consisted of only six items. Together, general and system-specific knowledge accounted for 34 percent of the variance in CPS performance.

A significant increase in domain-specific knowledge from pre- to post-test was observed for every subscale. The strongest effect was for declarative action knowledge (*t* = 8.16, *p* < 0.01, *d* = 0.70), with smaller effects observed for declarative system knowledge (*t* = 2.86, *p* < 0.01, *d* = 0.25) and procedural action knowledge (*t* = 2.33, *p* < 0.05, *d* = 0.20). Pre-post correlations were 0.83 (*p* < 0.01) for declarative system knowledge, 0.49 (*p* < 0.01) for declarative action knowledge, and 0.54 (*p* < 0.01) for procedural action knowledge.

#### An Integrative Path Model

**FIGURE 4:**
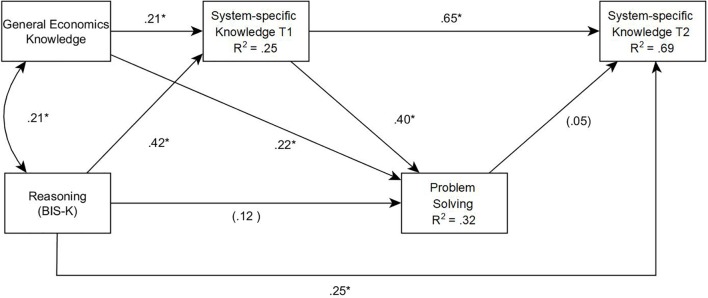
Study 1: Path model for problem solving performance in Tailorshop with knowledge and reasoning as predictors. χ^2^(1) = 0.347, *p* = 0.556, Comparative Fit Index (CFI) = 1.000. Values with ^∗^ are significant at the 5% level.

In a second step, we tested our theoretical model via path analysis. Reasoning and general economics knowledge were assumed to be correlated exogenous variables influencing the generation of hypotheses and the acquisition of system-specific knowledge during instruction and exploration, and thus also the amount of system-specific (prior) knowledge measured at time T1. We also assumed direct paths from reasoning, general economics knowledge and system-specific prior knowledge (T1) to control performance, and tested whether reasoning, domain-specific prior knowledge (T1) and problem-solving performance influence system-specific knowledge measured after controlling the system (T2). The resulting model is presented in **Figure 4**.

The path model reflects and extends the results above. System-specific prior knowledge (T1) was significantly influenced by the two correlated exogenous variables, indicating the importance of general domain knowledge, and especially of reasoning, for generating and testing hypotheses in the *Tailorshop* simulation. System-specific prior knowledge (T1) was influenced by learning processes during the instructions and, for a part of the sample, during system exploration. A total of 25.4% of the variance was explained by the two exogenous variables. General economics knowledge (β = 0.22, *p* < 0.01) and system-specific prior knowledge (T1; β = 0.40, *p* < 0.01) also had direct effects on control performance. Reasoning ability, meanwhile, had no direct effect (β = 0.12, *p* = 0.12), but a strong indirect effect on problem solving performance as mediated by prior knowledge. The total amount of explained variance in problem solving performance was 32%. Finally, system-specific knowledge after controlling the system (T2) primarily depended on system-specific prior knowledge (T1; β = 0.65, *p* < 0.01) as well as reasoning (β = 0.25, *p* < 0.01). Remarkably, while control performance and acquired system knowledge (T2) were substantially correlated (*r* = 0.46, *p* < 0.01), the direct path from control performance to acquired system-specific knowledge (T2) was not significant (β = 0.05, *p* = 0.35). Overall, 68.6% of the variance was explained.

### Discussion

Both intelligence and prior knowledge were shown to be important predictors of performance controlling a complex system. Some qualifications, however, must be made to this conclusion. First, it is not general intelligence that has predictive power for problem solving success in *Tailorshop;* instead and as expected, it is the primary factor reasoning, and more specifically numerical reasoning. This underscores the importance of finding the right level of symmetry between predictor and criterion in order to estimate their true relationship (Wittmann, 1988). Second, the correlation between reasoning and problem solving performance was mediated through prior knowledge; reasoning had no direct influence on problem solving performance. This finding is in line with the results of the meta-analysis by Schmidt et al. (1986; Schmidt, 1992), which showed that the relationship between intelligence and job performance is nearly completely mediated by task-related knowledge. This may indicate that persons with higher reasoning ability have used their ability to accumulate more domain knowledge in the past. The strong relationship between reasoning and general economics knowledge supports this account. An alternative explanation is that high reasoning ability helps people transfer their general domain knowledge to the specific situation, i.e., by deriving good hypotheses about the unknown system from their general theoretical knowledge about the corresponding domain. System-specific knowledge measured after controlling the system (T2) depends primarily on prior knowledge and reasoning. Therefore, controlling a complex system can be described as a knowledge acquisition process, providing evidence for Cattel’s investment theory (Cattell, 1987). Assuming that the system has ecologically validity, this finding also indicates that system-specific knowledge measured after controlling a complex system is a powerful predictor of external criteria.

The study was limited to the computer-simulated system *Tailorshop*, a microworld mainly developed by psychologists. The scenario is realistic in that it captures many psychologically relevant features of complex real-life problems, but its ecological validity as a model for a real business environment is limited. For example, real company executives spend more than 80% of their time communicating orally (e.g., Mintzberg, 1973; Kotter, 1982), a demand which was not implemented in the simulation (see Süß, 1996).

A final but important qualification to the study’s results concerns reasoning in the context of knowledge. System-specific knowledge was consistently the best single predictor of problem solving success in *Tailorshop*, while general domain knowledge in economics significantly predicted additional variance. System-specific knowledge was made up of two independent predictors, declarative system knowledge and declarative action knowledge. Our study found no evidence of the dissociation between verbalized knowledge and control performance repeatedly reported by Broadbent and colleagues (Broadbent et al., 1986; Berry and Broadbent, 1988; see Berry and Dienes, 1993). *Tailorshop* is a more complex and realistic system than those used by Broadbent and colleagues. Both factors might have strongly motivated people to make use of their preexisting knowledge, i.e., to formulate explicit hypotheses for controlling the system rather than following a trial-and-error approach that would result in the acquisition of implicit knowledge.

## Study 2

The aim of the second study was to replicate and extend the findings presented so far. Study 2 differed from Study 1 in two important ways. First, we used the artificial world simulation *FSYS* (Wagener, 2001), which simulated a forestry company. Although *FSYS* has a rich semantic embedding and all the characteristics of complex problems, *FSYS* was developed with the aim of reducing the impact of previous knowledge of the simulated domain (i.e., general forestry knowledge) on problem solving performance. Therefore, *FSYS* can be classified as a CAS. Second, we included WMC as a further predictor. WMC is a more basic construct than reasoning and whether it is a better (i.e., more symmetrical) predictor of CPS performance than reasoning is an open question (see Zech et al., 2017). Thus, we were interested in whether one of the two constructs had incremental validity in predicting CPS performance beyond the other construct.

### Method

#### Participants

One hundred fifty-nine students from the University of Magdeburg participated in the second study, which was originally conducted to evaluate a complex problem solving training (for details, see Kretzschmar and Süß, 2015), in 2010/2011.^7^ In the present analyses, we used the full sample but excluded all non-native German speakers (*n* = 7) due to the high language requirements of the intelligence test. The mean age was 23.99 years (*SD* = 4.43), and 50% were female. Participants received course credit for their participation or took part in a book raffle. Participants were informed about the content of the study, the voluntary nature of participation and their ability to withdraw at any point, and that anonymity was guaranteed. All subjects provided informed consent.

#### Materials

##### Problem solving

**FIGURE 5:**
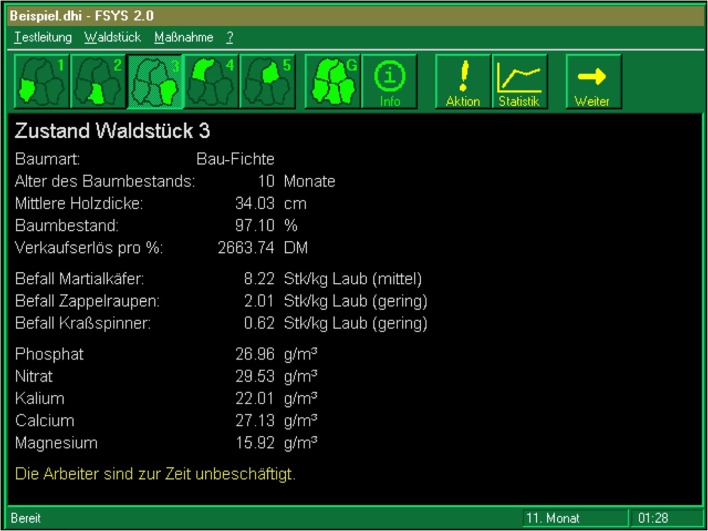
Screenshot of the exploration phase of FSYS system as applied in Study 2.

We used version 2.0 of the microworld *FSYS* (Wagener, 2001). *FSYS* was developed on the basis of Dörner et al.’s (1983) theoretical framework for complex problem solving (Dörner, 1986). It is a microworld with 85 variables connected via linear, exponential, or logistic relations. The goal was to manage five independent forests in order to increase the company’s value (i.e., planting and felling trees, fertilizing, pest control, etc.). Participants were first given an introduction to the program and had an opportunity to explore the system. They then managed the forest company for 50 simulated months. We used the company’s total capital (i.e., an aggregated score of the five independent forests) at the end of the simulation as the performance indicator (SKAPKOR; see Wagener, 2001). Although *FSYS* simulates a forestry enterprise, the impact of prior knowledge was reduced by using abstract names for tree species, pests, fertilizer etc., and providing essential information about the artificial foresting world via an integrated information system. Previous studies have shown that *FSYS* has incremental predictive validity beyond general intelligence with regard to occupational (Wagener and Wittmann, 2002) and educational (Stadler et al., 2016) performance indicators. **Figure 5** provides a screenshot of *FSYS*.

##### Intelligence

A short version of the BIS Test was used to assess intellectual abilities (Jäger et al., 1997). We specifically focused on reasoning and perceptual speed. Nine tasks were applied for each operation, balanced over the three content areas (i.e., figural, verbal, numerical; see **Figure 1**). These 18 tasks were administered according to the test manual. As in Study 1, the tasks were aggregated in order to build scales for each operation (i.e., reasoning, perceptual speed) or content (i.e., figural intelligence, verbal intelligence, numerical intelligence). An indicator for general intelligence was built by aggregating the 18 tasks in a balanced way, as described in the test handbook. Please note that the reliability of the two operative scales was lower than in Study 1; the construct validity of the three content scales and the measure of general intelligence were also reduced because no memory or creativity tasks were used. This limits the interpretability of the BIS content scales and the comparability of the results of the two studies.

##### Working memory

Working memory capacity was assessed with three tasks from the computerized test battery by Oberauer et al. (2003). The *numerical memory updating* (adaptive) and *reading span* (non-adaptive) tasks measured the simultaneous storage and processing functions of working memory, whereas the *dot span* task (also named *spatial coordination*; adaptive) primarily measured the coordination function. Moreover, each content category (i.e., figural, verbal, numerical) was represented by one task. A global score for WMC was calculated by aggregating the three equally weighted total task scores.

##### Knowledge

A questionnaire to assess general forestry knowledge as a measure of preexisting domain knowledge was developed for the purpose of this study^8^. It covered forestry knowledge in the subdomains of tree species, soils, nutrients, damage to a forest, and silviculture. An example question was: “Which tree is not a conifer?” The 22 multiple-choice items were scored dichotomously. Four items were excluded due to poor psychometric properties (i.e., a low item-total correlation). The remaining 18 items were aggregated to form a global sum score.

To assess system-specific knowledge about *FSYS*, we used Wagener’s (2001) knowledge test about the microworld. The 11 multiple-choice items addressed system and action knowledge across all relevant areas of *FSYS*. For example: “A forest is infested by vermin XY. Which procedure would you apply?” In order to limit the number of questions, we did not differentiate between different types of knowledge. Therefore, we used a sum score as a global indicator of system-specific knowledge.

#### Procedure

Participants took part in two sessions each lasting about 2.5 h. All testing was done in groups of up to 20 persons at the university computer lab. The first session comprised tests of intelligence and WMC. In the second session, participants completed tests of general forestry knowledge, complex problem solving, and system-specific knowledge. In contrast to Study 1, system-specific knowledge was assessed only once, after participants had worked with the CPS scenario (similar to Wagener, 2001). As the study was originally designed as an experimental training study (see Kretzschmar and Süß, 2015), the procedure differed slightly between the two experimental groups. About half of the participants completed the second session the day after the first session. The other half participated in a CPS training in between and completed the second session about 1 week after the first session.

### Results

We will first present results for individual groups of predictors of CPS performance before integrating the results into a combined path model. Due to the original study design (i.e., exclusion criteria for the training, dropout from the first session to the second), up to 24% of the data for the knowledge tests and the CPS scenario were missing. We used the full information maximum likelihood (FIML) procedure to account for missing data. The smallest sample size in the analyses of individual groups of predictors was 116. The data are publicly available via the Open Science Framework^9^. See **Table 3** for descriptive statistics and the full correlation matrix.

**Table 2:** Multiple regression of problem solving performance on the operation, content, and total scales of the BIS.

	Speed	Mem.	Creat.	Reas.	*R*^2^_adj_	Verb.	Fig.	Num.	*R*^2^_adj_	g	*R*^2^
Study 1: *Tailorshop*	-0.16 (0.08)	-0.16 (-0.03)	0.16 (0.22^∗^)	0.43^∗^ (0.34^∗^)	0.15^∗^	0.04 (0.16)	-0.01 (0.11)	0.22^∗^ (0.25^∗^)	0.04^∗^	0.22 (0.22)	0.05
Study 2: *FSYS*	0.02 (0.19^∗^)	–	–	0.33^∗^ (0.34^∗^)	0.10^∗^	-0.18 (0.07)	0.38^∗^ (0.37^∗^)	0.17 (0.27^∗^)	0.16^∗^	0.33^∗^ (0.33^∗^)	0.10^∗^


*Beta weights in the first line; bivariate Pearson correlations in brackets in the second line. Speed, perceptual speed; Mem., memory; Creat., creativity; Reas., reasoning; Verb, verbal intelligence; Fig., figural intelligence; Num, numerical intelligence. Values with ^∗^ are significant at the 5% level.*

**Table 3:** Study 2: Means, standard deviations, and correlations.

Variable	*M*	*SD*	1	2	3	4	5	6	7	8	9	10	11
(1) BIS: g	0.04	0.50											
(2) BIS: Speed	0.04	0.60	0.82**										
(3) BIS: Reasoning	0.04	0.57	0.88**	0.47**									
(4) BIS: Verbal	0.08	0.60	0.71**	0.69**	0.60**								
(5) BIS: Figural	0.02	0.62	0.84**	0.69**	0.71**	0.47**							
(6) BIS: Numerical	0.01	0.69	0.78**	0.67**	0.68**	0.35**	0.46**						
(7) WMC	0.12	2.10	0.55**	0.41**	0.52**	0.33**	0.45**	0.48**					
(8) Know: General	8.35	2.74	0.08	0.00	0.13	0.04	0.03	0.09	0.02				
(9) Know: Specific	5.32	1.94	0.36**	0.17	0.41**	0.20*	0.35**	0.23*	0.22*	0.19*			
(10) CPS	57.59	22.51	0.33**	0.19*	0.34**	0.07	0.37**	0.27**	0.32**	0.16	0.51**		
(11) Age	23.99	4.43	-0.34**	-0.21**	-0.34**	-0.23**	-0.36**	-0.17*	-0.31**	0.24*	-0.17	-0.21*	
(12) Gender	0.50	0.50	-0.17*	-0.05	-0.20*	0.13	-0.09	-0.35**	-0.06	-0.18	-0.20*	-0.10	-0.08


*^∗^ Indicates *p* < 0.05; ^∗∗^ indicates *p* < 0.01. *M* and *SD* are used to represent mean and standard deviation, respectively. BIS, Berlin Intelligence Structure Test; WMC, working memory capacity; Know: General, general forestry knowledge; Know: Specific, system-specific knowledge; CPS, complex problem solving performance (*FSYS*).*

#### Complex Problem Solving, Intelligence, and Working Memory

The results of two multivariate regressions of *FSYS* performance scores on the BIS operative and content scales, respectively, are summarized in **Table 2** (lower half, correlations in brackets). The results for operation abilities are similar to those from the first study, with reasoning the only significant predictor (β = 0.33, *p* < 0.01). However, figural intelligence was the only statistically significant predictor among the content scales (β = 0.38, *p* < 0.01). This seems plausible given that *FSYS* displays important information graphically rather than numerically (e.g., diagrams showing the forestry company’s development). However, a large amount of information is also presented numerically, meaning that numerical reasoning should exert an influence as well. Taking the cell level of the BIS into consideration: Numerical reasoning (Cronbach’s α = 0.66) became similarly strongly associated with *FSYS* control performance (*r* = 0.37, *p* < 0.01; corrected for unreliability *r* = 0.46) as figural reasoning (Cronbach’s α = 0.72; *r* = 0.36, *p* < 0.01; corrected for unreliability *r* = 0.42). Verbal reasoning (Cronbach’s α = 0.51) remained unassociated with *FSYS* performance (*r* = 0.02, *p* = 0.82). In contrast to Study 1, the content scales accounted for a slightly larger share of the variance in *FSYS* (16%) than the operation scales (10%). General intelligence (BIS-g) had a.33 (*p* < 0.01) correlation with problem solving performance.

Next, we compared the impact of reasoning and WMC as predictors of success in *FSYS*. Both predictors exhibited an almost equal and statistically significant zero-order correlation (*r*_BIS-R.__FSYS_ = 0.34, *p* < 0.01; *r*_WMC.__FSYS_ = 0.32, *p* < 0.01). In hierarchical regressions, each explained a similar but non-significant amount of incremental variance over and above the other predictor (Δ*R*^2^_BIS.K_ = 0.02; Δ*R*^2^_WMC_ = 0.02). The total explained variance was 12.2% (adjusted). In summary, working memory did not increase the statistical significance of the multiple correlation when entered as a second predictor.

#### Complex Problem Solving and Knowledge

General forestry knowledge was not significantly correlated with *FSYS* performance (*r* = 0.16, *p* = 0.09). Thus, the (non-)impact of prior domain knowledge in *FSYS* was similar as in previous studies (*r* = 0.13; Wagener, 2001), emphasizing how the impact of prior knowledge depends on the specific type of microworld (i.e., CRS in Study 1 vs. CAS in Study 2). The correlation between system-specific knowledge (measured after working on *FSYS*) and *FSYS* performance was *r* = 0.51 (*p* < 0.01).

#### An Integrative Path Model

In line with our assumptions about the relations among the predictor and criterion variables and building upon the results of the first study, we constructed a path model to integrate our findings. Perceptual speed from the BIS Test was excluded from the analyses because it was not significantly associated with any endogenous variable when controlling for reasoning. Prior general forestry knowledge was also omitted from the path model for the same reason.

**FIGURE 6:**
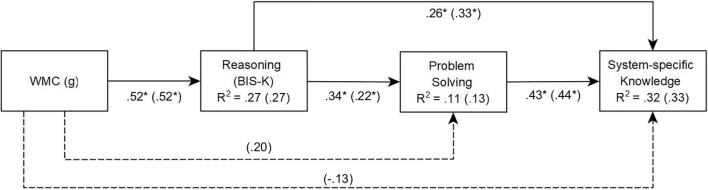
Study 2: Path Model A for problem solving performance and system-specific knowledge in *FSYS*, predicted by reasoning and working memory capacity (WMC). Fit for model A (without dashed lines): χ^2^(2) = 4.538, *p* = 0.10, Comparative Fit Index (CFI) = 0.977. Path model B (saturated) with dashed lines and values in brackets. Values with ^∗^ are significant at the 5% level.

In the first model (**Figure 6**, Model A), working memory had a direct influence on reasoning but not on *FSYS* control performance and system-specific knowledge. In this model [χ^2^(2) = 4.538, *p* = 0.10, CFI = 0.977, SRMR = 0.038], control performance (β = 0.34, *p* < 0.01) and acquired system-specific knowledge about the microworld *FSYS* (β = 0.26, *p* < 0.01) were significantly influenced by reasoning. The total amount of explained variance for control performance and system-specific knowledge were 11% and 32%, respectively.

In a second (fully saturated) model (**Figure 6**, Model B: dashed lines and coefficients in brackets), direct paths from working memory to *FSYS* control performance and system-specific knowledge were added. In this model, working memory had a small but non-significant direct effect on control performance (β = 0.20, *p* = 0.09), i.e., the effect of working memory is primarily based on its shared variance with reasoning. Furthermore, WMC functioned as a suppressor when it came to predicting system-specific knowledge. In other words, despite the positive zero order correlation between the two variables (see above), the direct path from WMC to system-specific knowledge was negative (β = -0.13, *p* = 0.19), while the impact of reasoning on system-specific knowledge slightly increased (β = 0.33, *p* < 0.01). On the other hand, the path from working memory to system-specific knowledge was statistically non-significant, and the explained variance in system-specific knowledge did not significantly increase [Δ*R*^2^ = 0.012, *F*(1,148) = 2.663, *p* = 0.46].

### Discussion

The general findings of Study 1 with regard to the impact of intelligence on CPS performance could be replicated in Study 2. However, as Study 2 was conducted with a different microworld with different cognitive demands (e.g., less relevance of prior knowledge), the results differed somewhat compared to those of Study 1.

With regard to intelligence, reasoning was again the strongest and sole predictor of CPS performance. Because general intelligence (g) was operationalized substantially more narrowly than in Study 1, the results for reasoning and g were comparable. These findings highlight the effect of the specific operationalization of intelligence selected. If intelligence is broadly operationalized, as proposed in the BIS (see Study 1), the general intelligence factor is not equivalent to reasoning (aka fluid intelligence; see also Carroll, 1993; McGrew, 2005; Horn, 2008) and different results for g and for reasoning in predicting CPS performance can be expected (see e.g., Süß, 1996). With regard to the content facet, *FSYS* shared the most variance with figural intelligence. However, the cell level of the BIS provided a more fine-grained picture: figural reasoning was just as highly correlated with *FSYS* performance as numerical reasoning. Although Study 1 and Study 2 must be compared with caution (i.e., due to different operationalizations of the BIS scales, see **Figure 1**, and limited BIS reliability on the cell level), it is clear that different CPS tests demand different cognitive abilities. At the same time, these findings highlight the importance of the Brunswik symmetry principle (Wittmann, 1988; Wittmann and Süß, 1999). A mismatch between predictor and criterion (e.g., figural reasoning and *Tailorshop* performance in Study 1; or numerical intelligence and *FSYS* performance in Study 2) substantially reduces the observed correlation (for another empirical demonstration in the context of CPS, see Kretzschmar et al., 2017). Ensuring that the operationalizations of the constructs are correctly matched provides an unbiased picture of the association across studies (Zech et al., 2017).

Working memory capacity was strongly related to reasoning and largely accounted for the same portion of variance in problem solving success as reasoning; it did not explain substantial variance over and above reasoning. These results complement the mixed pattern of previous findings, in which working memory explained CPS variance above and beyond intelligence (Wittmann and Süß, 1999), was the only predictor of CPS variance when simultaneously considering figural reasoning (Bühner et al., 2008), but did not explain CPS variance above and beyond reasoning (Greiff et al., 2016). In our view, there is little unique criterion variance to explain because the predictors are highly correlated. Even small differences in operationalization or random fluctuations can make one or the other predictor dominate (for a different view, see Zech et al., 2017).

Preexisting knowledge (i.e., general forestry knowledge) did not contribute to problem solving success. This finding highlights the importance of the CPS measurement approach selected. Whereas *Tailorshop* was developed as a complex real life-oriented simulation in which prior domain knowledge plays a substantial role, *FSYS* was developed with the aim of reducing the influence of prior knowledge (Wagener, 2001). Therefore, in addition to the distinction between microworlds and MCS, the differential impact of prior knowledge in terms of semantic embedding has to be considered when examining the validity of CPS (e.g., the effects might differ for CRS vs. CAS, as in the present study). It should be noted that in Stadler et al.’s (2015) meta-analysis, a study featuring FSYS (in which prior knowledge has no impact) and a study involving a virtual chemistry laboratory (in which prior knowledge has an effect; see Scherer and Tiemann, 2014) were both classified as single complex system studies. As a substantial portion of the variance in CPS performance in semantically embedded microworlds can be attributed to prior knowledge, the question arises as to whether a more fine-grained classification of the CPS measures in Stadler et al.’s (2015) meta-analysis would have resulted in different findings. In summary, the heterogeneity of different CPS measurements makes it difficult to compare studies or conduct meta-analyses (some would say impossible, see Kluwe et al., 1991).

## General Discussion

The presented studies had two main goals. First, we wanted to investigate the predictive validity of differentiated cognitive constructs for control performance in complex systems. Second, we were interested in how preexisting general knowledge and system-specific prior knowledge contribute to successful system control.

Both studies clearly demonstrate that intelligence plays an important role in control performance in complex systems. This is in contrast to former claims in early CPS research that problem solving success in complex, dynamic, partially intransparent systems is not at all correlated with intelligence test scores (e.g., Kluwe et al., 1991). Our results point to several explanations for prior failures to find positive correlations. First, previous studies used only a single problem solving trial, meaning that the performance criterion presumably was not satisfactorily reliable. Second, several previous studies did not differentiate between different aspects of intelligence, but used a measure of general intelligence. In our studies, however, general intelligence (g) as conceptualized in the BIS and operationalized with the BIS Test was not a good predictor of control performance. Instead and as was expected, the second-order construct of reasoning, and more specifically numerical reasoning, had the strongest relationship with success in the complex real-world oriented system (*Tailorshop*), while figural and numerical reasoning had the strongest relationship with success in the complex artificial world problem (*FSYS*). However, whether g and reasoning are distinguishable from each other (Carroll, 1993), and thus also whether the two differ in predicting CPS performance, depends on the level of generality, i.e., the broadness of the operationalization of g.

Our results are in line with the first Berlin study (Süß et al., 1993a,b) and several other studies using the *Tailorshop* system and other CRSs focusing on ecological validity (e.g., Wittmann and Süß, 1999; Kersting, 2001; Leutner, 2002; Rigas et al., 2002; Ryan, 2006; Danner et al., 2011), and were confirmed in Stadler et al.’s (2015) meta-analysis.

### Is There Evidence for a New Construct ‘Complex Problem Solving Ability’?

The two presented studies, however, are limited to one microworld each, and do not answer broader questions regarding generalizability. In particular, the convergent validity of microworlds was not addressed, but this question is essential for postulating *complex problem solving ability* as a new ability construct.

The following criteria must be considered in justifying a new ability construct (cf., Süß, 1996, 1999): (1) temporal stability, (2) a high degree of generality (i.e., the construct can be operationalized across different tasks, showing convergent validity), (3) partial autonomy in the nomological network of established constructs (i.e., the shared performance variance in different tasks cannot be explained by well-established constructs), and (4) evidence for incremental criterion validity compared to established constructs. In this section, we briefly review the empirical results regarding the existence of a unique CPS construct. We focus on CPS research utilizing CRS (i.e., microworlds with semantic embeddings)^10^.

The 1-year stability of CRS performance in the Berlin study (see Süß, 1996) was *r* = 0.49, which is substantial, but much lower than that for the intelligence constructs. The temporal stability of the BIS scales ranged from 0.65 for creativity to 0.90 for reasoning. In addition, the time-stable performance variance was explained completely by intelligence and prior knowledge (Süß, 1996). To the best of our knowledge, no results on temporal stability for other CRS and temporal stability for aggregated scores based on different CRS are currently available.

Wittmann et al., (1996; Wittmann and Süß, 1999; Wittmann and Hattrup, 2004) investigated the convergent validity of CRS. Wittmann et al. (1996) applied three different CRS (*PowerPlant*, *Tailorshop*, and *Learn*!), the BIS Test and domain-specific knowledge tests for each system to a sample of university students. The correlations of the CRS were significant but rather small (0.22–0.38), indicating low convergent validity^11^. However, because the reliability of each CRS was substantially higher than their intercorrelations, substantial system-specific variance has to be assumed. Performance on each of the three systems was predicted by reasoning and domain-specific prior knowledge to a substantial degree. In a structural equation model with a nested-factor BIS model (Schmid and Leiman, 1957; Gustafsson and Balke, 1993) as predictor, the CPS g-factor with two performance indicators for each of the three systems (i.e., the CPS ability construct) was predicted by general intelligence (β = 0.54), creativity (0.25) and reasoning (0.76), whereas perceptual speed and memory did not contribute to prediction (Süß, 2001)^12^. In this model, reasoning, though orthogonal to general intelligence, was the strongest predictor of the complex problem solving ability factor. Almost all of the variance could be explained by the BIS, putting the autonomy of the CPS construct into question.

In sum, there is no evidence for a new ability construct based on CRSs. This, however, does not mean that this kind of research cannot provide important new insights into CPS processes (see Süß, 1999), and that CPS performance cannot predict real-life performance beyond psychometric intelligence measures to a certain extent (e.g., Kersting, 2001; Danner et al., 2011).

Kersting (2001) predicted police officers’ job performance over 20 months on the basis of intelligence (short scales for reasoning and general intelligence from the BIS Test), CPS performance (two simulations, including *Tailorshop*), and acquired system-specific knowledge (measured after controlling the system). In a commonality analysis (Kerlinger and Pedhazur, 1973), 24.9% of job performance variance was explained. The strongest specific predictor was intelligence (7.3%; reasoning and general intelligence at about the same level); CPS performance and system-specific knowledge explained 3.9 and 3.0% of the overall criterion, respectively. The largest share of the variance was confounded variance between intelligence and system-specific knowledge (24.9%). In comparison to our first study, both intelligence scales had reduced predictive validity due to lower reliabilities. However, this study shows that exploring and controlling CRS must be considered a learning process. Acquired system knowledge represents invested intelligence (i.e., crystallized intelligence) and was a small but additional predictor of real-life performance beyond intelligence. This provides that ecological-valid complex systems can additionally predict external criteria, and are useful learning and training tools for acquiring domain-specific knowledge.

## Part II: Review and Critique of the Minimally Complex System (MCS) Approach

The research presented and discussed in the first part of the paper focuses on CRSs. From the beginning, CRS research was criticized for numerous reasons, including the lack of a formal description of the system, the lack of an optimal solution as an evaluation criterion for subjects’ behavior and performance, the uncontrolled influence of prior knowledge, low or unknown reliability of the scores, and low or even non-existent convergent validity and predictive validity with respect to relevant external criteria (for summaries, see e.g., Funke, 1995; Süß, 1996; Kluge, 2008a). Therefore, the MCS approach (Greiff et al., 2012) was developed to overcome the limitations of former microworlds. The MCS approach is remarkably prominent in recent CPS research, which may be a consequence of the higher reliability and validity such systems are assumed to have in comparison to CRS (e.g., Greiff et al., 2015b). Consequently, some might argue that research on CPS performance based on CRS, as presented in the first part of the paper, is less reliable and informative. However, whether the MCS approach is really a superior alternative to studying problem solving in complex situations remains up for debate.

The MCS approach updates and further develops ideas that have been present since the beginning of CPS research. Funke (1993) suggested artificial dynamic systems as a research tool based on systems of linear equations. Buchner and Funke (1993) proposed the theory of finite state automata as a tool for developing CPS tasks. Applying this, Kröner (2001; Kröner et al., 2005), for example, implemented *MultiFlux*, which simulates a fictitious machine, within the finite-state framework. This idea was further developed into MCS, e.g., *Genetics lab* (Sonnleitner et al., 2012) and *MicroDYN* (Greiff et al., 2012). Generally, about 9–12 artificial world tasks, tiny systems with up to three exogeneous and three endogenous variables each, are applied in three phases: (1) free system exploration, (2) knowledge acquisition (i.e., assessment of acquired system knowledge), and (3) knowledge application (i.e., assessment of action knowledge). The required testing time is less than 5 min for each minimal system. Each system provides three scores, one for each of the above-mentioned phases, which are then used to form three corresponding knowledge scales. According to our knowledge taxonomy, Phase 2 measures declarative system knowledge (i.e., relations between variables), while Phase 3 measures procedural action knowledge (i.e., system interventions in order to achieve a given goal). The items in these two subtests are similar to the items in the arrows task and the system-states task of the *Tailorshop* knowledge test. Whereas each item in the MCS scales refers to a different minimal system, all items in the *Tailorshop* knowledge test refer to the same system. Nevertheless, the MCS tasks are very similar to each other and implement only a small number of CPS characteristics, giving the subtests high internal consistencies. Specifically, all minimal systems can be fully explored with the simple strategy “vary one thing at a time” (VOTAT; e.g., Vollmeyer et al., 1996) or the closely related strategy “vary one or none at a time” (Beckmann and Goode, 2014; for additional distinctions see Lotz et al., 2017). No special training is necessary to learn these strategies. Instead, they can be learned by instruction or examples of correct and incorrect applications. On the other hand, these strategies are clearly not sufficient for exploring CRS, i.e., systems with *many* exogeneous variables, indirect and side effects, delayed effects, and eigendynamics, especially if the time for the task is limited or in real-time simulations (e.g., Bremer’s fire-fighter; Rigas et al., 2002). For the latter, the quality of one’s hypotheses, which is based on domain knowledge, is a necessary prerequisite for successfully exploring the system. In summary, the features of MCS measurements outlined here, along with further criticisms of this approach (e.g., Funke, 2014; Scherer, 2015; Schoppek and Fischer, 2015; Dörner and Funke, 2017; Funke et al., 2017; Kretzschmar, 2017), substantially narrow the validity of the MCS approach as an indicator of CPS.

On the other hand, the relevance of the MCS approach is shown by many studies that have modeled the internal structure of MCS tasks (e.g., Greiff et al., 2012; Sonnleitner et al., 2012), provided evidence that performance variance cannot be sufficiently explained by reasoning (e.g., Wüstenberg et al., 2012; Sonnleitner et al., 2013; Kretzschmar et al., 2016), found strong convergent validity as well as a lower correlation with a CRS (i.e., *Tailorshop*; Greiff et al., 2015b; for a different view, see Kretzschmar, 2017), and demonstrated incremental validity in predicting school grades beyond reasoning (e.g., Greiff et al., 2013b; Sonnleitner et al., 2013; for different results, see Kretzschmar et al., 2016; Lotz et al., 2016) and beyond a CRS task (Greiff et al., 2015b). MCS have been proposed as a tool for assessing 21st Century skills (Greiff et al., 2014) and were applied in the international large-scale study PISA to assess general problem-solving skills (OECD, 2014). They have further been proposed as training tools and evaluation instruments for these skills (e.g., Greiff et al., 2013a; Herde et al., 2016). This begs the question: how strong is the empirical evidence? Are these far-reaching conclusions and recommendations justified?

Studies provide support for the psychometric quality, especially the reliability, of the MCS approach, although scale building and some statistics have been criticized (Funke et al., 2017; Kretzschmar, 2017). Only one study so far has attempted to compare MCS and CRS. In it, Greiff et al. (2015b) argued that MCS had a higher validity than *Tailorshop* in predicting school grades. The knowledge scales assessed after exploring the system were used as predictors for the MCS. However, system-specific knowledge for *Tailorshop* after controlling the system was not assessed (Kretzschmar, 2017). Instead, control performance was used as a predictor of school grades. Control performance, however, is not a valid measure of acquired knowledge, as demonstrated in our first study. For this, additional tests are needed after controlling the system, conducted in both studies in this paper.

Minimally complex systems research also only sparingly addresses questions of construct validity related to the measures and the conclusions (i.e., generalizability; see Kretzschmar, 2015). This concerns the operationalization of CPS characteristics (i.e., the construct validity of the MCS), which was addressed in more detail above. However, limitations also exist concerning the choice of the additional instruments applied in validation studies. The construct validity of many instruments is considerably limited, causing results to be overgeneralized (cf., Shadish et al., 2002). For example, operationalizing reasoning (i.e., fluid intelligence) with a single task (e.g., the Raven matrices; Wüstenberg et al., 2012; Greiff and Fischer, 2013) is not sufficient. Construct validity is also restricted if only one task is used to measure WMC (e.g., Bühner et al., 2008; Schweizer et al., 2013). Since Spearman’s (1904), work we know that task-specific variance can be reduced only through heterogeneous operationalizations of the intended constructs. The two studies reported in this paper show how strongly the relationship between intelligence and CPS performance varies depending on the generality level of the intelligence construct (see also Kretzschmar et al., 2017). The symmetry problem was demonstrated here for the BIS, but is also evident with regard to other hierarchical intelligence models, e.g., the Three Stratum theory (Carroll, 1993, 2005), the extended Gf-Gc theory (Horn and Blankson, 2005; Horn, 2008), and the Cattell-Horn-Carroll theory (CHC theory; McGrew, 2005, 2009). Süß and Beauducel (2011), therefore, classified every task of the most frequently used tests into the BIS, the three stratum theory, and the CHC theory to give a framework for this problem.

According to the BIS (Jäger, 1982), every intelligence task depends on at least two abilities (an operative and a content ability), i.e., every task relates to two different constructs. By extension, the interpretation in terms of only one ability is of limited validity due to unintended but reliable task-specific variance. It is either necessary to have several tasks for every construct and theory-based aggregation (Jäger, 1982, 1984) to reduce unintended variance, or the interpretation must be limited to a more specific conclusion (e.g., to numerical reasoning in our first study). The two studies presented here and many others show that these kinds of problems substantially influence the validity of conclusions in intelligence and problem solving research as well as in many other fields (Shadish et al., 2002).

In summary, the MCS approach provides solutions to psychometrics problems in CPS research, especially the reliability problem, but its validity as an indicator of CPS performance is substantially restricted. In our view, MCS are an interesting new class of problem-solving tasks, but provide few insights into complex real-world problem solving. Modifications of the MCS approach toward increased complexity (e.g., MicroFIN; Neubert et al., 2015; Kretzschmar et al., 2017) are a promising step in the right direction.

### Conclusion and Outlook

The primary aim of CPS research with CRSs (e.g., *Lohhausen*; Dörner et al., 1983) is ecological validity, i.e., “the validity of the empirical results as psychological statements for the real world” (Fahrenberg, 2017). In the past, many systems were “*ad hoc*” constructions by psychologists that had not been sufficiently validated, but this need not be the case. What is needed is interdisciplinary research in the form of collaboration with experts in the simulated domains. For example, Dörner collaborated with a business expert to develop *Tailorshop. Powerplant* was developed by Wallach (1997) together with engineers from a coal-fired power plant near Saarbrücken (Germany). *LEARN!*, a complex management simulator with more than 2000 connected variables, was originally developed by an economics research group at the University of Mannheim (Germany) as a tool for testing economic theories (Milling, 1996; Größler et al., 2000; Maier and Größler, 2000). In the version applied by Wittmann et al. (1996), participants have to manage a high-technology company competing with three others simulated by the computer. *ATC* (*Air Traffic Controller* Test; Ackerman and Kanfer, 1993) and *TRACON* (*Terminal Radar Air Control*; Ackerman, 1992) are simplified versions of vocational training simulators for professional air traffic controllers. The *Situational Awareness Real Time Assessment Tool* (*SARA-T*) was developed to measure the situational awareness of air traffic controllers working in the *NLR ATM Research Simulator* (*NARSIM*; ten Have, 1993), a system also used in expert studies (Kraemer and Süß, 2015; Kraemer, 2018). Finally, technological developments (e.g., video clips, virtual worlds; Funke, 1998) have enabled the development of complex systems that are much more similar to real-world demands than ever before, an opportunity that should be capitalized upon in psychological research (see Dörner and Funke, 2017).

In this line of research, the ecological validity of the simulated real-world relationships is essential and must be ensured. In addition, domain-specific prior knowledge is necessary to generate hypotheses for system exploration and system control. Valid measures of the amount, type, and structure of domain-specific prior knowledge, the knowledge acquisition processes, and the acquired knowledge are necessary for understanding and measuring CPS behavior and performance. In light of all this, this line of research can help us to understand how people face the challenge of dealing with complexity and uncertainty, identify causes of failure, and detect successful strategies for reducing complexity during problem solving (e.g., Dörner, 1996; Dörner and Funke, 2017), a laborious and time-consuming but important field of research in complex decision making (cf., Gigerenzer and Gaissmaier, 2011). The research strategy of restricting complex problem solving tasks to MCS, however, leads into a cul-de-sac.

## Ethics Statement

The studies were carried out in accordance with the ethical guidelines of the German Association of Psychology with informed consent from all subjects. Considering the time when the studies were conducted and the fact that the materials and procedures were not invasive, the studies were not approved by an ethical committee.

## Author Contributions

H-MS conceptualized the manuscript and conducted the first study. AK conducted the second study. H-MS and AK analyzed the data and drafted the manuscript in collaboration.

## Conflict of Interest Statement

The authors declare that the research was conducted in the absence of any commercial or financial relationships that could be construed as a potential conflict of interest.

## Footnotes

1 The correlation between complex real life-oriented systems and reasoning was not reported, nor was the effect of outliers on relationships other than that between CPS and g.

2 Participants only took the economics test in the first Berlin study, i.e., these data were assessed 1 year before all others reported here.

3 The first group could explore the system for 30 min on their own (exploration group), while the second group could study the system’s causal model for 30 min following standardized instructions (instructions group). The third group had no opportunity to acquire additional system-specific knowledge (control group). In this paper, we use the results for the full sample without considering the experimental variations. Experimental results and group-specific results are reported in Süß (1996).

4 The correlation with CPS was slightly higher (*r* = 0.27) for a conventional g-score based on the factor scores of the first unrotated factor (Jensen and Wang, 1994), i.e., 7.3% of CPS variance was explained.

5 According to the BIS, numerical reasoning is not a more specific ability but a performance based on reasoning and numerical intelligence (Jäger, 1982).

6 The correlation of CPS performance with figural reasoning was 0.26, and 0.24 with verbal reasoning.

7 A subsample was used in Kretzschmar and Süß (2015) to evaluate a CPS training. However, none of the relations between CPS and the variables used in the present study have been previously examined (for details, see the data transparency table at https://osf.io/n2jvy). Therefore, all analyses and findings presented here are novel.

8 We would like to thank Clemens Leutner for professional advice in developing the questionnaire.

9 https://osf.io/n2jvy

10 For a review focusing on CPS research applying the minimally complex systems (MCS) approach, see Kretzschmar and Süß (2015).

11 In the study of Ryan (2006) with 298 University students the intercorrelations of three scenarios, *Furniture Factory* (FF), *Taylorshop* (T) and *FSYS* (F), were also rather small but significant (*r*_FF,T_ = 0.30, *r*_FF,F_ = 0.27, *r*_T,F_ = 0.10; Stankov, 2017).

12 The structural equation model by Süß (2001) is copied in Wittmann and Hattrup (2004) as **Figure 6**. This model was built in two steps: First, BIS and CPS-g were modeled separately. Specific CPS factors for the three systems were not modeled because only two indicators were available for each system. Instead, the errors of the two indicators in each system were allowed to correlated as system-specific variance. Second, the five BIS factors (g and the four operative abilities) were used to predict CPS-g. Fit statistics for the final model are not valid because the loadings of both measurement models were optimized in the first step.
